# Identification of chalcone isomerase family genes and roles of *DnCHI1* in flavonoid metabolism in *Dendrobium nobile*

**DOI:** 10.3389/fpls.2025.1687738

**Published:** 2025-11-14

**Authors:** Ling-ling Chang, Yu-xin Zhou, Ling-rong Wang, Lei Liu, Zhen-jian Li

**Affiliations:** State Key Laboratory of Tree Genetics and Breeding, Research Institute of Forestry, Chinese Academy of Forestry, Beijing, China

**Keywords:** *Dendrobium nobile*, chalcone isomerase, flavonoid biosynthesis, DnCHI1, MYB

## Abstract

*Dendrobium nobile* is a prized orchid species with both medicinal and ornamental values, known for accumulating flavonoids that contribute to its bioactivity and flower pigmentation. Chalcone isomerase (*CHI*), a key enzyme in the flavonoid biosynthetic pathway, catalyzes the conversion of chalcone to naringenin, thereby promoting the production of anthocyanins and other flavonoids. In this study, we present the first systematic analysis of the *CHI* gene family in *D. nobile*, classifying them into two subfamilies. Expression profiling revealed that *DnCHI1*, a type IV *CHI*, is highly expressed in petals, suggesting a potential role in anthocyanin metabolism. Heterologous transient overexpression assays showed that *DnMYB90* significantly downregulated endogenous *PeCHI* expression and reduced anthocyanin accumulation, whereas *DnCHI1* overexpression resulted in a 15-fold increase in anthocyanin content. Dual-luciferase reporter and yeast one-hybrid assays further confirmed that *DnMYB90* acts as a transcriptional repressor of *DnCHI1*. These results provide new insights into the regulatory module in anthocyanin biosynthesis in *D. nobile*, highlighting the functional divergence of *CHI* genes and their interaction with MYB transcription factors.

## 1 Introduction

*Dendrobium nobile* Lindl. (Orchidaceae) is a perennial herbaceous species in the genus *Dendrobium*. The *Chinese Pharmacopoeia* records its rich content of bioactive compounds, including polysaccharides, alkaloids, and flavonoids, underpinning its pharmacological effects. Its vibrant floral coloration and distinctive morphology also make it an attractive ornamental species (Xu et al., 2022a) and an important parental line in *Dendrobium hybrida* breeding programs (Meng et al., 2025).

Flavonoids, a major class of plant secondary metabolites, are widely distributed across plant organs. In flowering plants, floral color, a key ornamental trait, is determined primarily by pigments such as carotenoids, flavonoids, and alkaloids (Qiu et al., 2023). Among these, anthocyanins, a subclass of flavonoids, are the predominant determinants of floral color diversity (Sun et al., 2015). The flavonoid biosynthetic pathway originates from the phenylpropanoid pathway, in which phenylalanine is sequentially converted to *p*-coumaroyl-CoA, a central intermediate that can enter either the flavonoid biosynthetic pathway or the monolignol biosynthetic pathway. Within the flavonoid pathway (Ge et al., 2023), chalcone synthase (*CHS*) and chalcone isomerase (*CHI*) catalyze the first two committed steps, generating essential precursors for downstream anthocyanin biosynthesis (Chao et al., 2021). In *Hevea brasiliensis*, for example, the overexpression of *HbCHI2* significantly upregulates downstream genes such as anthocyanin synthase (*ANS*), flavonoid 3′-hydroxylase (*F3′H*), and leucoanthocyanidin reductase (*LAR*), thereby enhancing flavonoid accumulation (Wu et al., 2025a).

*CHI* is the second enzyme in the flavonoid biosynthetic pathway (Yin et al., 2019; Nan et al., 2022). It catalyzes the intramolecular cyclization of 2′,4,4′,6′-tetrahydroxychalcone (produced by *CHS*) into (2*S*)-flavanone (Kang et al., 2014). While this reaction can occur spontaneously under physiological conditions, *CHI* accelerates it by approximately 10^7^-fold, greatly boosting flavonoid output in plant cells. Phylogenetic and functional analyses classify plant *CHI*s into four types (I–IV) (Luo et al., 2025). Types I and II exhibit true *CHI* activity, catalyzing chalcone to naringenin, which is essential for flavonoid biosynthesis and anthocyanin biosynthesis (Ni et al., 2020). Type I *CHI*s are widely distributed across vascular plants, including *Aquilaria sinensis* (Ding et al., 2021), bryophytes, ferns, and most angiosperms (Ni et al., 2020). Type II *CHI*s are largely confined to legumes, although some occur in basal land plants (Cheng et al., 2018). Type III and IV *CHI*s lack catalytic residues and therefore demonstrate no chalcone-cyclizing activity (Dastmalchi and Dhaubhadel, 2015). Type IV *CHI*s, also known as chalcone isomerase-like proteins (*CHIL*s), instead regulate flavonoid biosynthesis (Zhao et al., 2020; Wolf-Saxon et al., 2023). *CHIL*s are unique to land plants (Jiang et al., 2015) and remain among the least characterized components of the *Arabidopsis* flavonoid pathway. For instance, eggplant *SmCHI2* is preferentially expressed in pigmented tissues and promotes anthocyanin accumulation (Zhou et al., 2023), while *DcCHI4* from *Dracaena cambodiana* enhances flavonoid production when overexpressed in *Nicotiana benthamiana* (Zhu et al., 2021). Such findings highlight the regulatory importance of *CHIL* proteins.

In orchids, flavonoid biosynthesis is tightly controlled by transcription factors, particularly the MYB family, which modulates structural gene expression and thereby floral pigmentation. MYB proteins are classified into four groups based on the number and arrangement of MYB repeats (R) in their conserved domains: 1R-MYB, R2R3-MYB, R1R2R3-MYB, and 4R-MYB. Of these, R2R3-MYB proteins are predominant regulators of anthocyanin biosynthesis (Yan et al., 2021; Yang et al., 2022; Li et al., 2015). In *Phalaenopsis-type Dendrobium*, *DhMYB2* and *DhbHLH1* enhance petal anthocyanin accumulation by activating *DhDFR* and *DhANS* (Li et al., 2017). Similarly, the transient overexpression of R2R3-MYB genes *RcPAP1* and *RcPAP2* from Cattleya in *Phalaenopsis* induced purplish-red pigmentation in previously white tepals (Li et al., 2020). In *Dendrobium officinale*, *DoMYB5* and *DobHLH24* directly bind to the promoters of *DoCHS* and *DoDFR*, positively regulating anthocyanin biosynthesis (Yang et al., 2023). A recent genomic analysis of *D. nobile* identified 125 *MYB* genes, classified into 26 subgroups, with *DnMYB90* assigned to subgroup S4, suggesting a potential role in flavonoid regulation (Wu et al., 2024). In addition, the systematic characterization of the *DnCHI* gene family revealed two type IV *CHI*s (*DnCHI1* and *DnCHI3*). Subsequent qRT-PCR analysis revealed that *DnCHI1* expression was the highest in petals. Under dual-luciferase reporter and yeast one-hybrid assays, we confirmed that *DnMYB90* binds to the *DnCHI1* promoter, implicating it in the transcriptional regulation of anthocyanin metabolism. These findings advance our understanding of the regulatory network governing floral pigmentation in *D. nobile* and provide a framework for further research into flavonoid biosynthesis in orchids.

## 2 Materials and methods

### 2.1 Identification of *CHI* gene family members from *D. nobile*

The genome assembly, protein sequences, and annotation files of *D. nobile* were obtained from the National Center for Biotechnology Information (NCBI; https://www.ncbi.nlm.nih.gov/, accessed on April 7, 2025) under accession number GCA_022539455.1. *CHI* genes in *D. nobile* were identified by performing a BlastP search against the *D. nobile* protein database using characterized *Arabidopsis thaliana CHI* amino acid sequences from the TAIR database (https://www.arabidopsis.org/) as queries (Zhang et al., 2017). Candidate *CHI* genes were selected with an e-value cutoff of 1e^−5^. The *CHI* family-specific Pfam accession (PF02431) was retrieved from the Pfam database (http://pfam.xfam.org/), and its HMM profile was downloaded (Potter et al., 2018). Using the Simple HMM Search tool in TBtools, the *D. nobile* genome was systematically screened for potential *CHI* family members. Candidate sequences were validated via protein domain analysis using NCBI-CDD (https://www.ncbi.nlm.nih.gov/Structure/cdd/wrpsb.cgi) and SMART (http://smart.embl-heidelberg.de), and those lacking the conserved *CHI* domain were excluded. Six *CHI* genes were ultimately identified and named *DnCHI1*–*DnCHI6* according to their chromosomal locations. The physicochemical properties of each protein were estimated using ProtParam (http://web.expasy.org/protparam/), and their subcellular localization was predicted using Plant-mPloc (http://www.csbio.sjtu.edu.cn/bioinf/plant-multi/) (Chou and Shen, 2010; Artimo et al., 2012).

### 2.2 Phylogenetic analysis of the *DnCHI* genes

A phylogenetic tree was constructed using MEGA 11.0 with the neighbor-joining (NJ) algorithm, analyzing *CHI* protein sequences from *A. thaliana*, *Oryza sativa*, *Glycine max*, and *D. nobile* to investigate the evolutionary relationships among *CHI* genes. Bootstrap analysis was conducted using 1,000 replicates under default parameters. The resulting tree was exported in Newick format and visualized using the iTOL online tool (https://itol.embl.de/).

### 2.3 Conserved motif and gene structure analysis

The conserved motifs of the *DnCHI* gene family were analyzed using the MEME suite (http://meme-suite.org/) with the following parameters: Maximum motifs = 10 and optimum motif width = 6–50 amino acids (Bailey et al., 2009). Gene structures (exon–intron organization) were determined by aligning coding sequences (CDS) with their corresponding genomic DNA sequences in TBtools. Motif distribution and gene structure were visualized using TBtools visualization modules to enable comparative analysis among *DnCHI* members.

### 2.4 Analysis of *cis*-active elements of the *DnCHI* genes

The promoter regions (2,000 bp upstream of the ATG start codon) of all *DnCHI* gene family members were extracted using TBtools and analyzed for *cis*-acting elements using the PlantCARE online platform (http://bioinformatics.psb.ugent.be/webtools/plantcare/html/). The results were visualized using TBtools.

### 2.5 Plant materials and sample preparation

Mature *D. nobile* plants were collected from Chishui City, Guizhou, China, and cultivated under natural light conditions in the Chinese Academy of Forestry greenhouse (Beijing, China). Root, stem, leaf, and flower tissues were harvested at full bloom; immediately frozen in liquid nitrogen for 1 hour; and stored at −80°C. Three biological replicates were prepared for each tissue type.

### 2.6 Tissue-specific expression analysis

The frozen tissues of *D. nobile* were ground in liquid nitrogen, and total RNA was extracted using the DP441 RNA Extraction Kit (Tiangen, Beijing, China). RNA concentration and purity were measured using a NanoDrop™ One/OneC spectrophotometer (Thermo Fisher Scientific, Waltham, MA, USA), and integrity was assessed using agarose gel electrophoresis. First-strand cDNA was synthesized using the One-Step gDNA Removal and cDNA Synthesis SuperMix kit (TransGen Biotech, Beijing, China). Gene-specific primers (**Supplementary Table 1**) were designed using NCBI Primer-BLAST and validated using the TBtools Primer Check tool, with all primers synthesized by Sangon Biotech (Shanghai, China). The *DnUBQ1* gene was used as an internal reference based on a prior evaluation of housekeeping genes in *D. nobile* (Guo et al., 2012). qRT-PCR reactions were prepared according to the manufacturer’s protocol of TB Green^®^ Premix Ex Taq™ (Takara, Tokyo, Japan) and run on a LightCycler 480 System (Roche, Basel, Switzerland). Relative expression levels were calculated using the 2^−ΔΔCt^ method (Livak and Schmittgen, 2001). Each sample analysis included three biological replicates and four technical replicates.

### 2.7 Cloning of *DnCHI1* and *DnMYB90*

The reference sequences of *DnCHI1* (LOC110099164) and *DnMYB90* (KAI0514214.1) were retrieved from the NCBI database (https://www.ncbi.nlm.nih.gov). Gene-specific primers were designed using SnapGene 8.0 (**Supplementary Table 1**). Target fragments were amplified from petal cDNA templates using 2× TransStart^®^ FastPfu Fly PCR SuperMix (-dye) (TransGen Biotech, Beijing, China), and amplification products were verified using agarose gel electrophoresis. Positive bands were excised and purified using a gel extraction kit (GenBetter, Beijing, USA) and subsequently ligated into the pMD19-T cloning vector (Takara, Japan). The recombinant plasmids were transformed into *Escherichia coli* DH5α competent cells (Huayueyang, Beijing, China) using the freeze–thaw method. Positive clones were screened using colony PCR and confirmed by sequencing (Sangon Biotech, Shanghai, China).

### 2.8 Transient heterologous expression of *DnCHI1* and *DnMYB90* in *Phalaenopsis*

Given the lack of a reliable transient overexpression system for *D. nobile*, *Phalaenopsis sogo* Yukidian ‘V3’, which has a well-established transformation system, was used for heterologous transient expression (Li et al., 2020). The binary vector pCAMBIA1302 was linearized with *Nco*I and purified. Using SnapGene 8.0, primer pairs for vector construction (**Supplementary Table 1**) were used to construct recombinant plasmids p1302-*DnCHI1* and p1302-*DnMYB90*, which were transformed into *E. coli* DH5α, and single clones were selected for sequencing at Sangon Biotech (Shanghai, China). The sequencing-verified plasmids were introduced into *Agrobacterium tumefaciens* GV3101 (Huayueyang, Beijing, China). Positive colonies were selected using PCR and cultured in Luria-Bertani (LB) medium containing 20 mg/L rifampicin and 50 mg/L kanamycin. Bacterial cells were collected by centrifugation at optimal density and resuspended in infiltration buffer (10 mM MgCl_2_, 10 mM MES, and 200 µM acetosyringone). The suspensions were syringe-infiltrated into *P. sogo* Yukidian ‘V3’ petals. Following visible phenotype development, the relative expression levels of ABP genes were determined via qRT-PCR using published primers (**Supplementary Table 1**), with *PeActin* as the reference gene. All measurements included six biological replicates, each with four technical replicates. Anthocyanins were extracted from 0.5 g of petal tissue using 10 mL of acidic methanol containing 1% (v/v) HCl and incubated overnight at 4°C. After centrifugation, the supernatant was collected, and absorbance was measured at 530 and 657 nm using a spectrophotometer. Anthocyanin content (AT) was calculated using the formula AT (U/g) = A_530_ − 0.25 × A_657_ (Zhou et al., 2024). All assays were performed in triplicate.

### 2.9 Subcellular localization of *DnMYB90*

The coding sequence of *DnMYB90* (without a stop codon) was inserted into the GFP-tagged pCAMBIA1302 vector using seamless cloning (primers listed in **Supplementary Table 1**) to generate the 35S::*DnMYB90*-GFP construct (Jiang et al., 2023). Both the empty vector and 35S::*DnMYB90*-GFP were transformed into the *A. tumefaciens* strain GV3101. Positive transformants were cultured, resuspended, and infiltrated into the abaxial side of *N. benthamiana* leaves. After 48 hours of incubation, fluorescence signals were observed using a confocal laser scanning microscope (Zeiss LSM 880 Meta, Jena, Germany). Three independent biological replicates were performed for each construct.

### 2.10 Dual-luciferase assay

Genomic DNA was extracted using a rapid DNA extraction kit (Tiangen, Beijing, China). The *DnCHI1* promoter was amplified by nested PCR using genomic DNA and inserted into the pGreenII 0800-LUC reporter vector via homologous recombination. The *DnMYB90* coding sequence was cloned into the pGreenII 62-SK effector vector (primer sequences in **Supplementary Table 1**). Recombinant vectors were transformed into *A. tumefaciens* GV3101 (pSoup) and verified by bacterial PCR. The bacterial suspensions were co-infiltrated into different sites of the same *N. benthamiana* leaf to ensure internal comparability. After 48 hours under low-light conditions, LUC/REN ratios were quantified using the Dual-Luciferase^®^ Reporter Assay System (Promega, Madison, WI, USA) on a multimode plate reader, and fluorescence activity was visualized using the NightSHADE LB 985 imaging system (Berthold, Bad Wildbad, Germany). All assays included three biological replicates, each with three technical replicates.

### 2.11 Yeast one-hybrid assay

The *DnCHI1* promoter sequence was truncated into 150–350-bp fragments containing specific binding sites to avoid potential auto-activation. The truncated promoter fragments and the *DnMYB90* coding sequence were inserted into the pLacZi-2μ bait vector and pB42AD prey vector, respectively, via seamless cloning (primers listed in **Supplementary Table 1**). To preclude autoactivation and ensure experimental rigor, two control groups were established: Control 1 (*DnCHI1pro*::LacZi + Empty pB42AD) and Control 2 (empty pLacZi + AD-*DnMYB90*). Two independent constructs were generated for each combination and co-transformed into *Saccharomyces cerevisiae* strain EGY48 and plated on SD dropout medium (SD/-Trp/-Ura). After 2–3 days of inverted incubation at 28°C, single colonies were diluted, serially diluted, and spotted onto SD/-Trp/-Ura/Gal/Raf/X-Gal indicator plates. Both colony growth and blue coloration were subsequently recorded.

## 3 Results

### 3.1 Identification and physicochemical property analysis of the *DnCHI* genes

**Figure 1:**
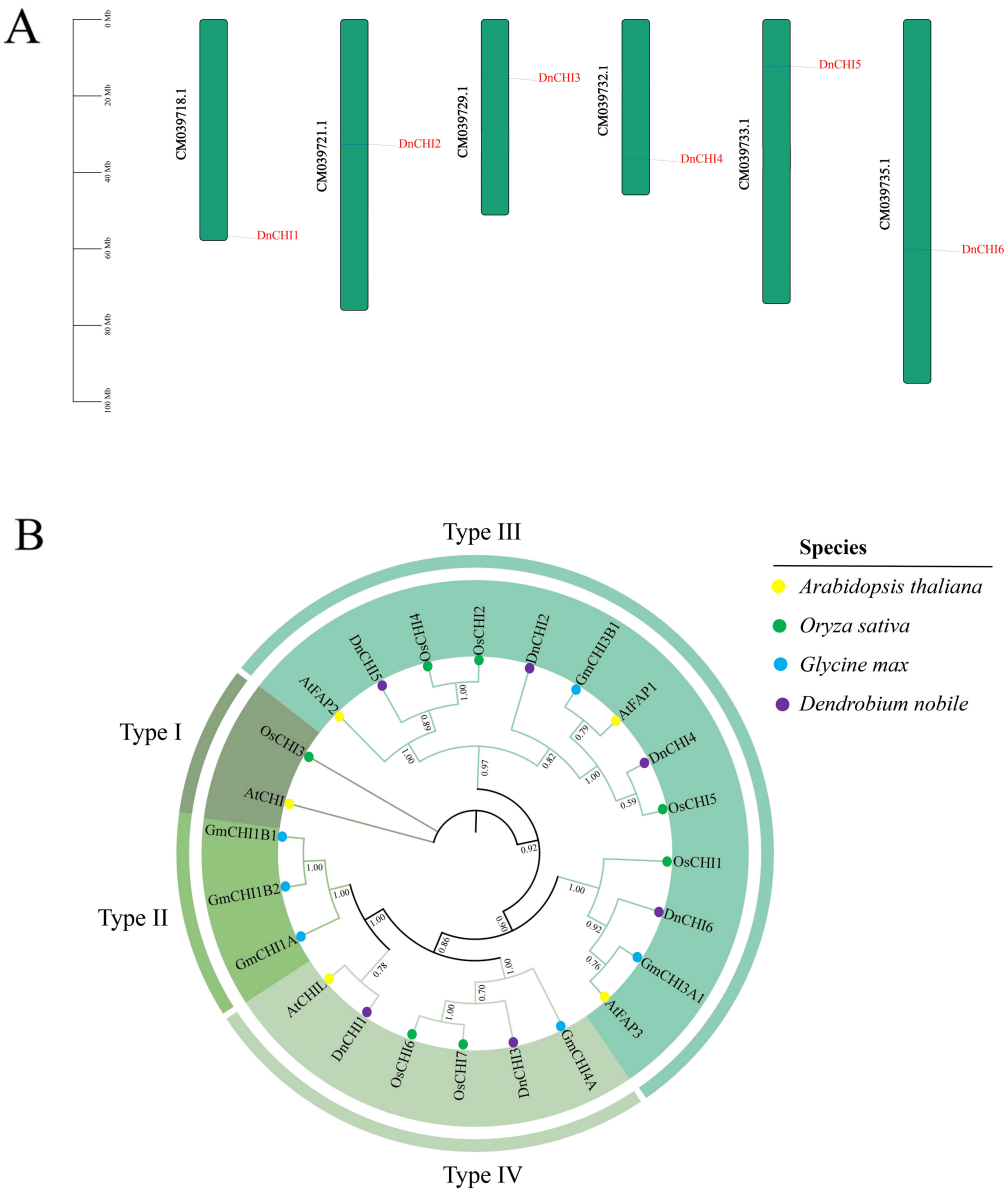
Chromosomal localization and phylogenetic analysis of *DnCHI* genes. **(A)** Distribution of *DnCHI* genes from *Dendrobium nobile* across six chromosomes. The scale on the left indicates chromosome length (in megabases, Mb). **(B)** Phylogenetic analysis of *CHI* genes among *Arabidopsis thaliana* (yellow circle), *Oryza sativa* (green circle), *Glycine max* (blue circle), and *D*. *nobile* (purple circle). The unrooted phylogenetic tree was constructed using the neighbor-joining (NJ) method in the MEGA X software with 1,000 bootstrap replicates.

Six *CHI* genes were systematically identified from the *D. nobile* genome and designated as *DnCHI1* to *DnCHI6* according to their chromosomal locations (**Figure 1A**). The *CHI* gene family members displayed considerable variation in physicochemical properties, with protein lengths ranging from 209 to 425 amino acids (*DnCHI5* being the longest and *DnCHI3* the shortest), molecular weights varying between 23,682.12 and 47,187.5 Da, and theoretical isoelectric points (pI) from 4.99 to 9.28. Instability indices varied from 35.1 to 45.23, aliphatic indices from 92.8 to 98.2, and the grand average of hydropathicity values from −0.161 to 0.047, with four members predicted as hydrophilic proteins. Subcellular localization predictions indicated predominant chloroplast localization for most *DnCHI* proteins, additional nuclear localization for *DnCHI2* and *DnCHI5*, and unique membrane localization for *DnCHI6* (**Supplementary Table 2**).

### 3.2 Phylogenetic analysis of the *DnCHI* genes

A phylogenetic tree was constructed using five *A. thaliana*, seven *O. sativa*, six *G. max*, and six *DnCHI* genes, revealing four distinct subfamilies (Types I–IV), with Type III containing the highest number of members (**Figure 1B**). Four *DnCHI* genes (*DnCHI2*, *DnCHI4*, *DnCHI5*, and *DnCHI6*) clustered within Type III, which, according to previous studies, can be further subdivided into three subgroups (*FPA1*, *FPA2*, and *FPA3*) involved in fatty acid metabolism. The Type IV subfamily included two *DnCHI* genes (*DnCHI1* and *DnCHI3*) that may encode key enzymes for flavonoid biosynthesis (Wang et al., 2022). No *D. nobile CHI* genes were classified within Type I or Type II subfamilies.

### 3.3 Conserved motif and gene structure analysis

**Figure 2:**
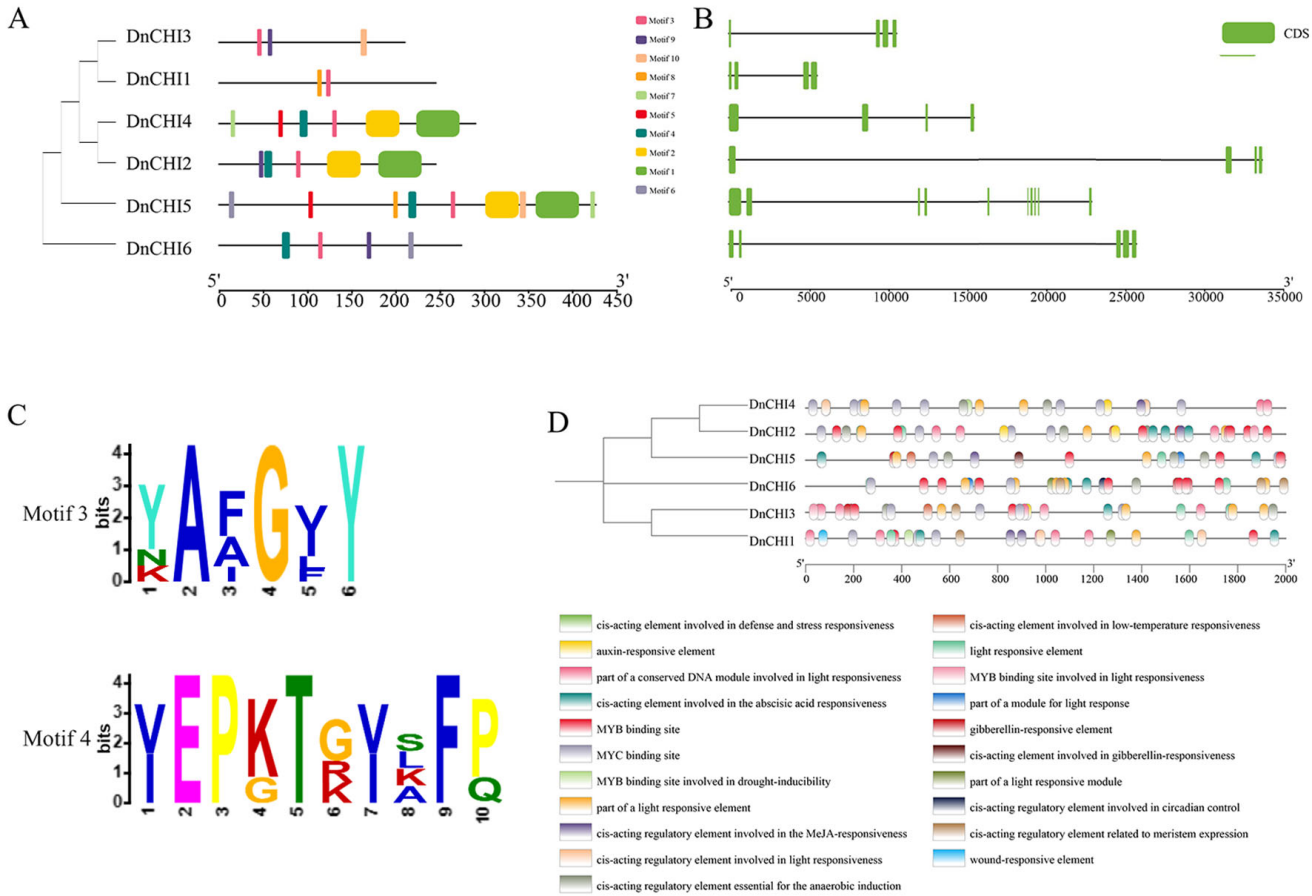
Conserved motif, gene structure, conserved domains, and *cis*-acting regulatory elements of the *DnCHI* genes. **(A)** Ten conserved motifs in the *DnCHI* genes are represented by different colored squares. **(B)** Exon and intron structures of *DnCHI* genes in *Dendrobium nobile*. Exons are shown as green squares and introns as gray lines. **(C)** Conserved motifs motif3 and motif4. **(D)** Promoter element sites, with colored rectangles representing distinct *cis*-acting regulatory elements.

MEME-based analysis identified 10 conserved motifs (motifs 1–10) with copy numbers ranging from 2 to 9 per protein (**Figure 2A**). Motif 3 was universally present across all *DnCHI*, while motif 4 was exclusively found in Type III subfamily proteins (**Figure 2C**). The Type III subfamily exhibited significantly higher motif abundance compared with the two Type IV members, accompanied by distinct motif compositions and exon positional arrangements between subfamilies, suggesting higher motif abundance compared with Type IV members, accompanied by distinct motif compositions and exon positional arrangements between subfamilies, suggesting greater structural conservation within Type III and potential functional divergence during evolution. Within the Type III, despite quantitative differences in motif numbers, similar compositional patterns and spatial distributions were observed, indicating conserved functional roles among these evolutionarily related proteins.

During gene family evolution, introns and exons play distinct roles in gene expression and regulation. The six *DnCHI* family members in *D. nobile* contained 4–10 exons, with *DnCHI5* possessing the highest number (10 exons). Four genes (*DnCHI3*, *DnCHI1*, *DnCHI4*, and *DnCHI2*) each contain four exons, while *DnCHI6* has five exons (**Figure 2B**). Genes within the same subgroup generally displayed similar exon numbers, with intron positions remaining relatively conserved throughout evolution.

### 3.4 *cis*-regulatory element analysis of *DnCHI* promoters

*cis*-Acting elements within gene promoter regions are key regulators of transcriptional activity and participate extensively in diverse biological processes (Wittkopp and Kalay, 2012). An analysis of the promoter regions of the *DnCHI* genes revealed *cis*-acting elements grouped into four major functional categories: regulation of plant growth and development, hormone responsiveness, stress responses, and transcription factor binding (**Figure 2D**). Most *DnCHI* promoters contained elements responsive to abscisic acid, auxin, methyl jasmonate, gibberellin, and light signaling, as well as binding sites for MYB and MYC transcription factors. A notable exception was *DnCHI4*, whose promoter lacked abscisic acid-responsive elements and MYB binding sites, which were conserved in all other members.

### 3.5 Tissue-specific expression analysis of *DnCHI1* and *DnCHI3* genes

**Figure 3:**
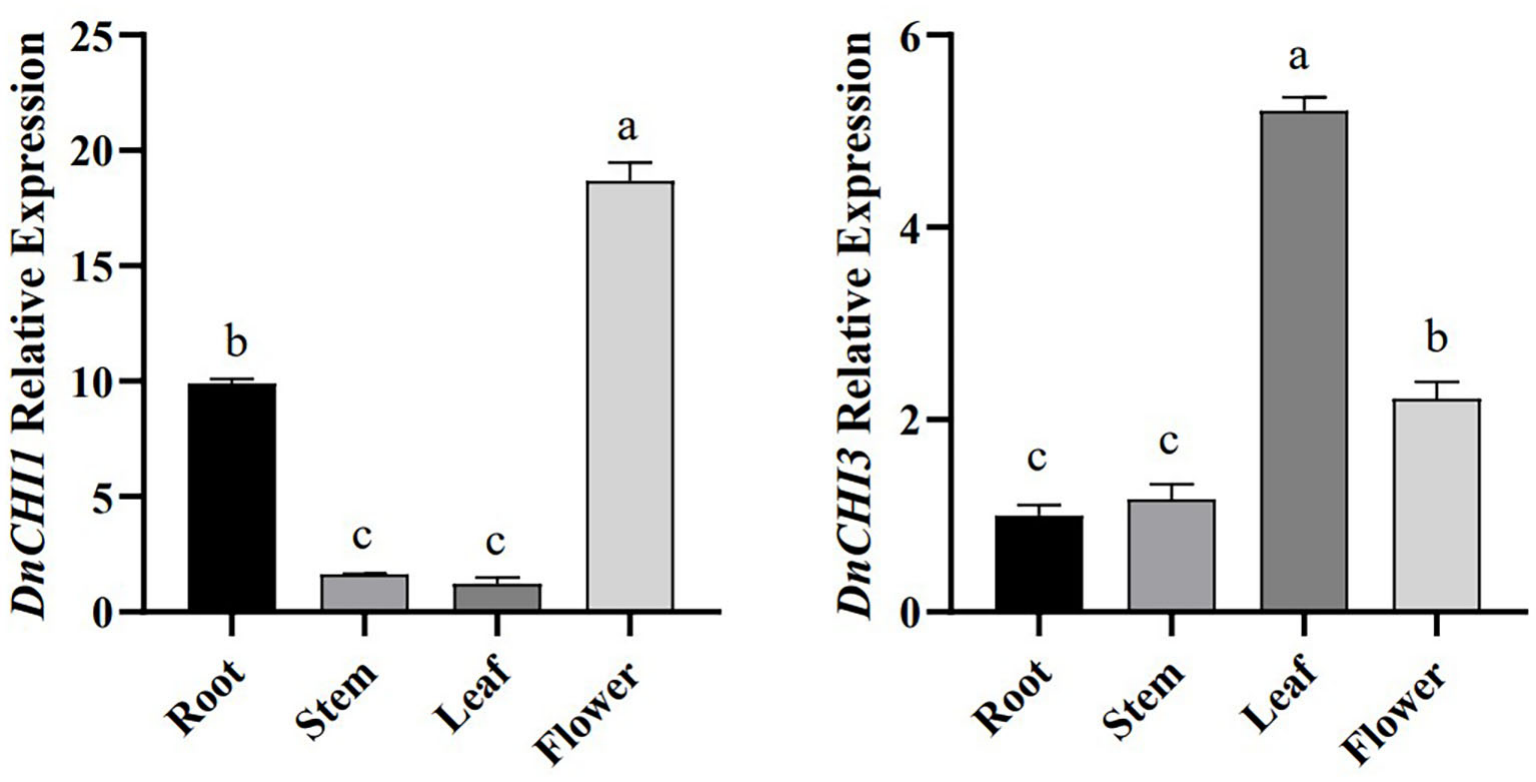
Relative expression levels of *DnCHI1* and *DnCHI3* in different tissues of *Dendrobium nobile*. Different lowercase letters indicate significant differences (p < 0.05).

qRT-PCR analysis revealed distinct expression profiles of *DnCHI1* and *DnCHI3* across roots, stems, leaves, and flowers of *D. nobile* (**Figure 3**). *DnCHI1* showed the highest transcript accumulation in petals, followed by roots, with minimal expression in stems and leaves. In contrast, *DnCHI3* exhibited predominant expression in leaves. The distinct tissue-specific expression patterns, together with a positive correlation between transcript abundance and secondary metabolite accumulation, suggest the potential involvement of type IV *DnCHI1* in floral pigment biosynthesis via secondary metabolic pathways.

### 3.6 Functional analysis of *DnCHI1* and *DnMYB90* through heterologous overexpression

**Figure 4:**
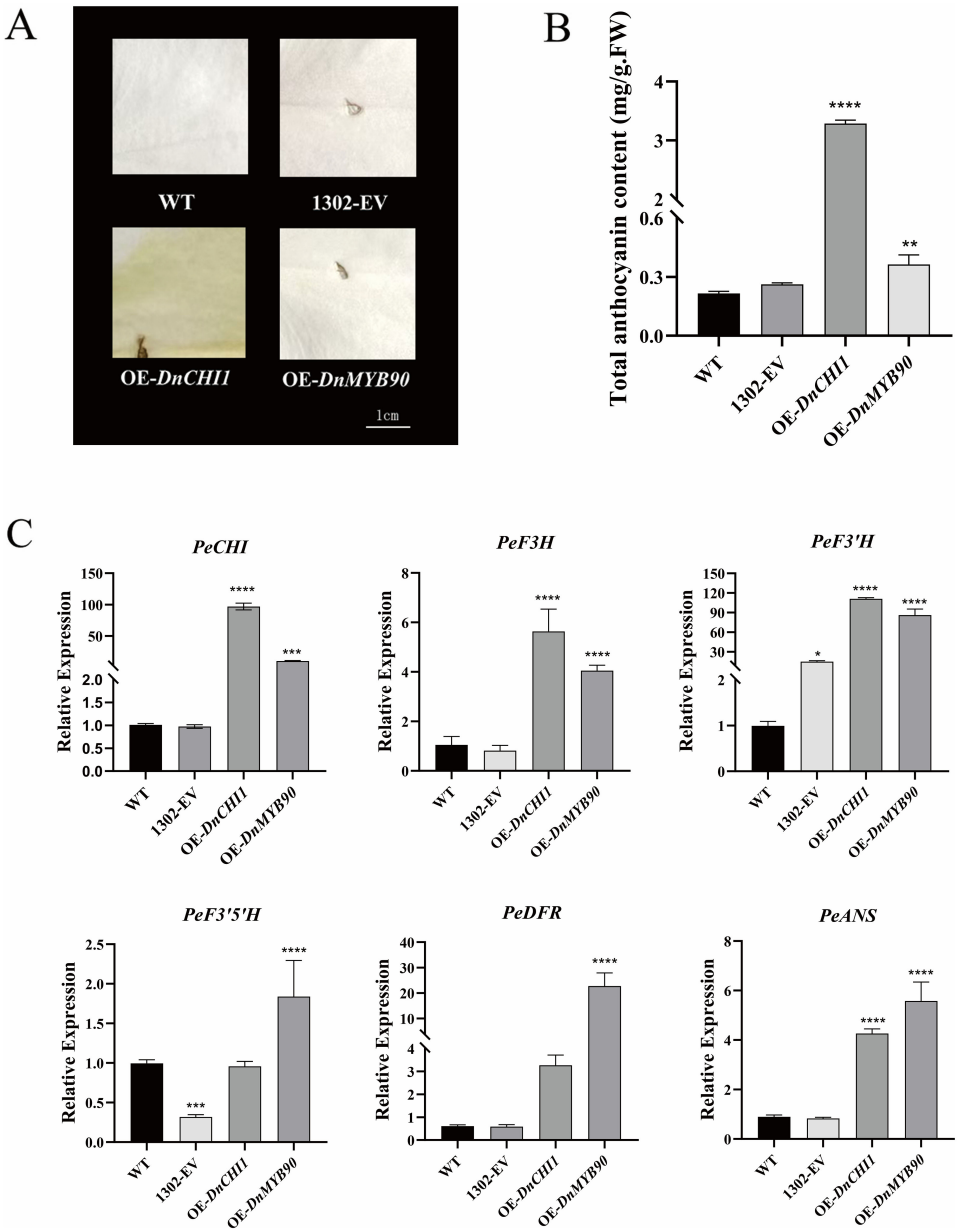
Transient overexpression of *DnCHI1* and *DnMYB90* in *Phalaenopsis* petals. **(A)** Flower phenotypes. WT, wild-type; 1302-EV, empty-vector control; OE-*DnCHI1* and OE-*DnMYB90*, overexpression lines. **(B)** Total anthocyanin content in transgenic lines. **(C)** Expression levels of anthocyanin biosynthesis-related genes. Error bars represent SEM of six biological replicates. Asterisks indicate statistical significance (*p < 0.05; **p < 0.01; ***p < 0.001; ****p < 0.0001). Scale bar = 1.0 cm.

The heterologous overexpression of *DnCHI1* and *DnMYB90* in *Phalaenopsis* petals induced distinct phenotypic changes. *DnCHI1*-overexpressing lines exhibited significant yellow pigmentation relative to controls, whereas *DnMYB90*-overexpressing plants displayed coloration comparable to empty-vector (pCAMBIA1302) controls (**Figure 4A**). Anthocyanin quantification revealed substantially higher pigment accumulation in *DnCHI1*-transformed petals compared with wild type, whereas *DnMYB90* overexpression resulted in moderate but significantly lower anthocyanin increases (**Figure 4B**).

qRT-PCR analysis confirmed effective transgene expression, with wild-type and empty-vector controls showing comparable expression levels. *DnCHI1* overexpression markedly upregulated flavonoid biosynthesis genes (*PeCHI*, *PeF3H*, *PeF3′H*, and *PeANS*), whereas *DnMYB90* enhanced *PeF3′H*, *PeF3′5′H*, and *PeANS* expression (111-fold and 85-fold increases in *PeF3′H* for *DnCHI1* and *DnMYB90* overexpressors, respectively), despite lower *PeCHI* expression in *DnMYB90* transformations (**Figure 4C**). Both transgenic systems consistently upregulated *DnANS* expression.

### 3.7 Nuclear localization of *DnMYB90*

**Figure 5:**
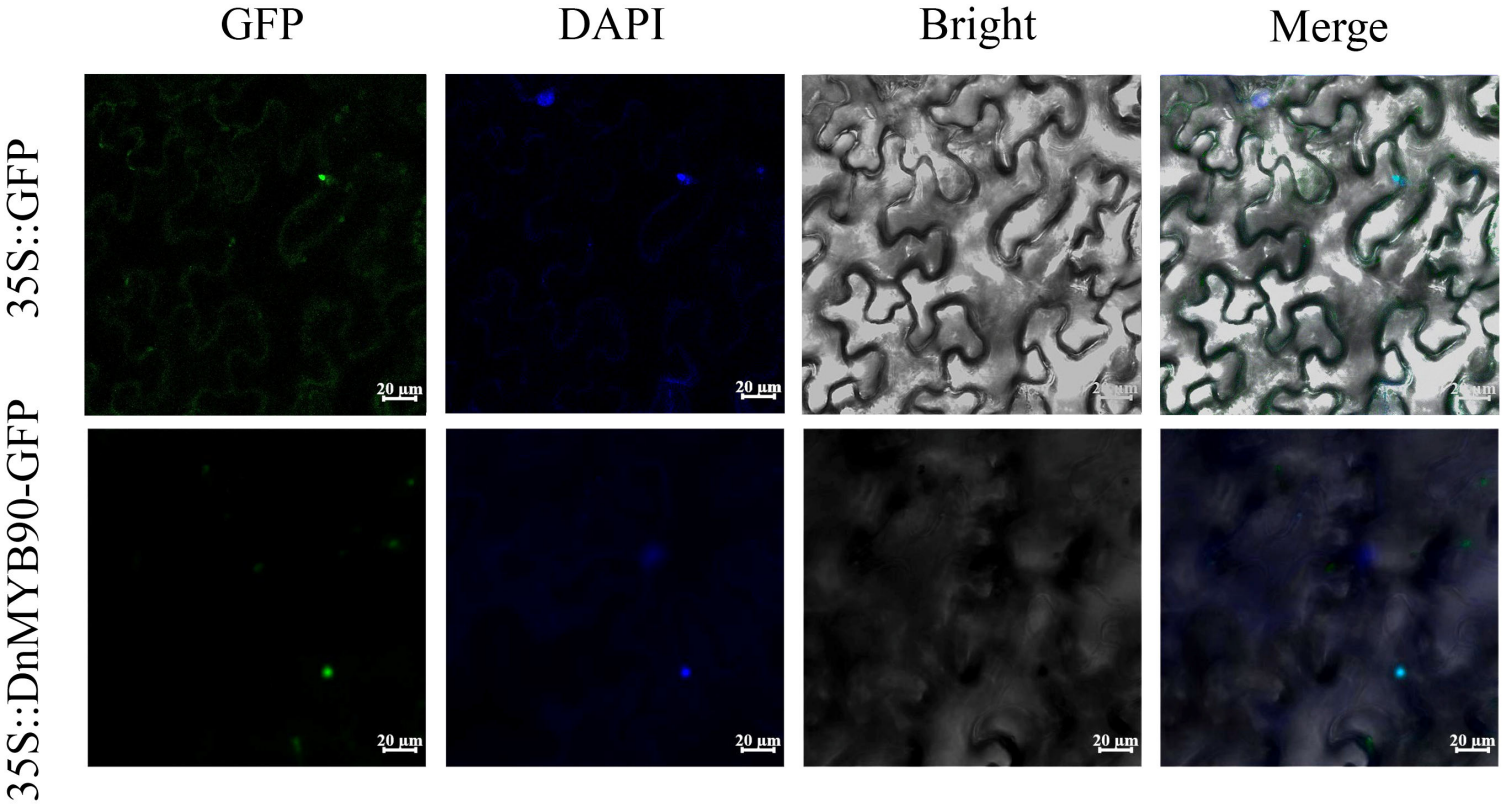
Subcellular localization of *DnMYB90*. GFP, green fluorescent protein fluorescence; DAPI, blue nucleus fluorescence; Merge: the superposition of green and blue fields. Bar = 20 μm.

Subcellular localization analysis provided insights into the functional properties of *DnMYB90*. In *N. benthamiana* leaf cells, GFP-tagged empty vectors exhibited fluorescence in both the nucleus and cytoplasm, whereas the 35S::GFP-*DnMYB90* fusion protein localized exclusively to the nucleus. These findings indicate that *DnMYB90* functions primarily within the nuclear compartment (**Figure 5**).

### 3.8 *DnMYB90* bound and inhibited the *DnCHI1* promoter

**Figure 6:**
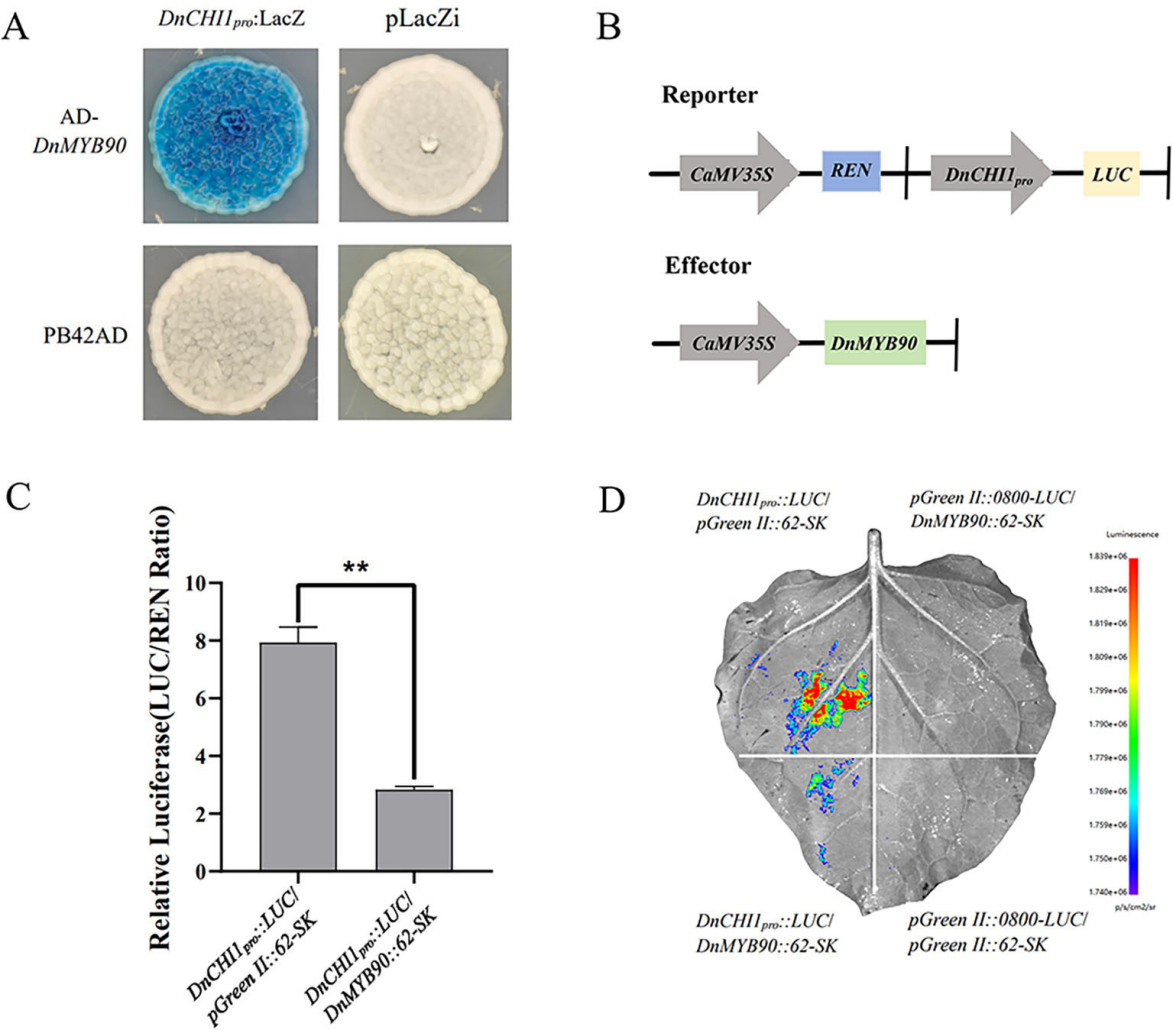
Regulatory effect of *DnMYB90* on *DnCHI1*. **(A)** Yeast one-hybrid analyses of the interaction between *DnMYB90* and the promoter of *DnCHI1*. **(B)** Schematic of reporter and effector constructs. **(C)** Dual-luciferase reporter assay showing repression of *DnCHI1* promoter activity by *DnMYB90*. Data represent mean ± SE (n = 3). Statistical significance was determined by Student’s t-test (**p < 0.01). **(D)** Representative image of a *Nicotiana benthamiana* leaf 3 days after co-infiltration.

The bioinformatics analysis of the *DnCHI1* promoter sequence (**Figure 2D**) identified conserved MYB transcription factor binding sites. Yeast one-hybrid assays confirmed a direct interaction between *DnMYB90* and the *DnCHI1* promoter region (**Figure 6A**). Dual-luciferase reporter assays (**Figures 6B–D**) demonstrated that *DnMYB90* significantly repressed *DnCHI1* promoter activity, thereby establishing a regulatory mechanism in which *DnMYB90* binds to and negatively regulates *DnCHI1*.

## 4 Discussion

*CHI* is a key enzymatic regulator in the flavonoid biosynthetic pathway, influencing both the rate and yield of flavonoid production in plants. In this study, six *DnCHI* genes with putative roles in flavonoid biosynthesis were identified in *D. nobile*. A classification system for *G. max CHI* genes (Type I–IV), first established by Dastmalchi and Dhaubhadel (2015), has since been applied to model plants such as *A. thaliana*, in which *AtCHI* represents Type I, *AtFAP1/2/3* belong to Type III, and *AtCHIL* is a Type IV gene (Ngaki et al., 2012). Subsequent studies have expanded *CHI* gene characterization across diverse species. For example, Wu et al. identified five *IbCHI* genes in *Ipomoea batatas* (Wu et al., 2025b) and reported 33 *CHI* family members across four *Gossypium* species, with expression patterns responsive to Fusarium wilt race 7 infection (Zu et al., 2019). In *Allium fistulosum*, Xu et al. (2022) demonstrated that Type IV *AfCHIL* enhances naringenin synthesis efficiency via functional interaction with *AfCHS* (Xu et al., 2022b). Phylogenetic analysis in this present study classified the six identified *DnCHI* genes in *D. nobile* into two distinct subfamilies: Type III (*DnCHI4*/*5*/*6*) and Type IV (*DnCHI1*/*3*), with no Type I or II members detected. Functional divergence was evident among subfamilies, with Type II members occurring exclusively in leguminous plants and Type III members typically associated with fatty acid biosynthesis. Although Type I *CHI*s functionally overlap with Type IV *CHI*s, they generally exhibit lower catalytic efficiency. In contrast, Type IV CHIs often act as enhancer-flavonoid producers (EFPs), promoting flavonoid biosynthesis (Luo et al., 2025; Ngaki et al., 2012). The absence of Type I and Type II *CHI* genes in *D. nobile* may reflect limitations in the current genome assembly or annotation; however, it more plausibly represents a distinct evolutionary trajectory unique to this species. The conserved structural features of these *DnCHI* genes suggest functional conservation with established orthologs, although experimental validation is required to confirm these predicted roles.

Distinct expression profiles were observed for *DnCHI1* and *DnCHI3*: *DnCHI*1 was predominantly expressed in petals, while *DnCHI3* showed higher transcript abundance in leaves. Similar spatial expression trends have been documented in other species, such as root-preferential *CHI* expression in *Scutellaria baicalensis* (Lam et al., 2021) and the leaf-dominant expression of *Camellia sinensis CsF3′H* (Wang et al., 2017). Given the established correlation between flavonoid content and biosynthetic gene activity, the findings suggest that *DnCHI1* is a strong candidate gene involved in petal-specific metabolic processes.

Flavonoids represent one of the most important classes of plant secondary metabolites, and *CHI* expression has been shown to enhance flavonoid content in multiple systems (Yin et al., 2019). The heterologous expression of onion *CHI* in Del/Ros1-overexpressing tomatoes substantially increased anthocyanin content in both peel and pulp (Lim and Li, 2017), and the overexpression of *CnCHI4* from camellia markedly promoted flavonoid biosynthesis in transgenic tobacco and camellia plants (Yu et al., 2022). In this study, a qRT-PCR analysis of *DnCHI1*-overexpressing and *DnMYB90*-overexpressing lines revealed markedly lower *PeCHI* expression in OE-*DnMYB90* compared with OE-*DnCHI1*, whereas downstream ABP genes (*PeF3′5′H*, *PeDFR*, and *PeANS*) exhibited higher expression in OE-*DnMYB90* plants. Results showed that OE-*DnMYB90* plants accumulated less anthocyanin than OE-*DnCHI1* plants. This apparent paradox suggests that the negative effect of the *DnMYB90*-mediated transcriptional repression of the upstream key gene *DnCHI1* may outweigh the positive effect of its partial upregulation of downstream genes. Supporting this view, Kriangphan et al. (2015) proposed that the low color intensity observed in the pink hybrid orchid *D.* ‘Sirinclassic’ can be attributed to the reduced expression of upstream ABP genes *CHI1* and *CHI2*, resulting in limited naringenin production and consequently lower pigment accumulation and color intensity. Furthermore, the yellow pigmentation observed in OE-*DnCHI1* plants suggests that the overexpression of *DnCHI1* may promote flavanol accumulation, potentially enhancing the species’ medicinal value. Future studies could use Liquid Chromatography-Mass Spectrometry (LC–MS) to quantify metabolite levels in both OE-*DnMYB90* and OE-*DnCHI1* plants to validate these hypotheses. The preliminary evidence from yeast one-hybrid and dual-luciferase assays supports the hypothesis that *DnMYB90* directly represses *DnCHI1*. However, we cannot exclude the possibility that *DnMYB90* also directly or indirectly regulates other genes in the anthocyanin biosynthetic pathway (Dubos et al., 2010), with these multifaceted regulations collectively shaping the observed phenotype.

The R2R3-MYB subfamily, closely associated with anthocyanin regulation, has been well characterized in orchids, including *Cymbidium goeringii* (104 members), *Cymbidium faberi* (102 members), *D. officinale* (101 members), and *Phalaenopsis equestris* (99 members) (Wang et al., 2018; Fan et al., 2020; Ke et al., 2021; Chen et al., 2022). SG4-clade MYB transcription factors in particular often act as repressors of the ABP via the downregulation of key biosynthetic genes (Chen et al., 2022). Based on a genome-wide phylogenetic and conserved motif analysis of the R2R3-MYB family in *D. nobile*, Wu et al. (2024) classified *DnMYB90* within this subgroup, suggesting that it may function analogously as a repressor. In *A. thaliana*, R2R3-MYBs such as MYB11 and MYB12 have been shown to activate *CHI* gene expression (Pandey et al., 2015; Liu et al., 2015), and the overexpression of *EsAN2* in tobacco upregulated the expression of key flavonoid biosynthetic genes, including *CHS*, *CHI*, and *ANS* (Huang et al., 2016). However, in *D. nobile*, the regulatory mechanisms of SG4-clade R2R3-MYBs on *CHI* expression remain poorly understood, and no prior study has provided direct evidence of MYB–type IV *CHI* promoter interaction. This study provides preliminary evidence through dual-luciferase and yeast one-hybrid assays, indicating that *DnMYB90* directly binds the *DnCHI1* promoter and represses its transcription in heterologous systems. These findings offer new insight into the regulation of anthocyanin biosynthesis in *D. nobile*. Nevertheless, given the complexity of the ABP regulatory network, *DnMYB90*-mediated repression of *DnCHI1* alone is unlikely to represent the sole control point. Further studies are needed to evaluate its broader effects on ABP genes, as well as to functionally characterize additional SG4-clade *DnMYB*s to determine whether such repressive regulation is a conserved feature in this orchid species.

## 5 Conclusions

Six *CHI* gene family members were identified in the *D. nobile* genome, which are clustered into two distinct phylogenetic subfamilies. Expression profiling revealed the predominant accumulation of type IV *DnCHI1* transcripts in petals, indicating a potential key role in anthocyanin metabolism. Heterologous transient overexpression in *Phalaenopsis* demonstrated that *DnMYB90* overexpression led to significantly lower *PeCHI* expression and reduced anthocyanin accumulation compared with *DnCHI1* transformants. Dual-luciferase reporter and yeast one-hybrid experiments confirmed that *DnMYB90* acts as a transcriptional repressor of *DnCHI1*, providing new mechanistic insights into the regulation of anthocyanin biosynthesis in orchids. These findings establish a framework for the further elucidation of the complex transcriptional networks governing floral pigmentation. To further elucidate the mechanistic role of *DnMYB90*, subsequent studies could employ electrophoretic mobility shift assays (EMSAs) to quantify its binding affinity to the *DnCHI1* promoter, together with chromatin immunoprecipitation (ChIP) assays to comprehensively identify target genes involved in anthocyanin biosynthesis. In addition, yeast two-hybrid assays may be applied to screen for transcription factors that interact with *DnMYB90* and potentially co-regulate anthocyanin biosynthesis.

## Data Availability

The original contributions presented in the study are included in the article/**Supplementary Material**. Further inquiries can be directed to the corresponding author.
